# Overexpression of V-type H^+^ pyrophosphatase gene *EdVP1* from *Elymus dahuricus* increases yield and potassium uptake of transgenic wheat under low potassium conditions

**DOI:** 10.1038/s41598-020-62052-5

**Published:** 2020-03-19

**Authors:** Yongbin Zhou, Yan Li, Xueli Qi, Rongbang Liu, Jianhui Dong, Weihuan Jing, Mengmeng Guo, Qinglin Si, Zhaoshi Xu, Liancheng Li, Chengshe Wang, Xianguo Cheng, Youzhi Ma, Ming Chen

**Affiliations:** 1 Institute of Crop Science, Chinese Academy of Agricultural Sciences (CAAS)/National Key Facility for Crop Gene Resources and Genetic Improvement, Key Laboratory of Biology and Genetic Improvement of Triticeae Crops, Ministry of Agriculture, Beijing, 100081 China; 2 Wheat Research Institute, Henan Academy of Agricultural Sciences, Zhengzhou, Henan 450002 China; 3 College of Agronomy, Northwest A&F University, Yangling, Shaanxi 712100 China; 4 Ministry of Agriculture Key Laboratory of Plant Nutrition and Nutrient Cycling, Institute of Agricultural Resources and Regional Planning, Chinese Academy of Agricultural Sciences, Beijing, 100081 China

**Keywords:** Genetic engineering, Molecular engineering

## Abstract

Lack of potassium in soil limits crop yield. Increasing yield and conserving potassium ore requires improving K use efficiency (KUE). Many genes influence KUE in plants, but it is not clear how these genes function in the field. We identified the V-type H^+^-pyrophosphatase gene *EdVP1* from *Elymus dahurica*. Gene expression analysis showed that *EdVP1* was induced by low potassium stress. Protein subcellular localization analysis demonstrated that EdVP1 localized on the plasma membrane. We overexpressed *EdVP1* in two wheat varieties and conducted K tolerance experiments across years. Yield per plant, grain number per spike, plant height, and K uptake of four transgenic wheat lines increased significantly compared with WT; results from two consecutive years showed that *EdVP1* significantly increased yield and KUE of transgenic wheat. Pot experiments showed that transgenic plants had significantly longer shoots and roots, and higher K accumulation in shoots and roots and H^+^-PPase activity in shoots than WT under low K. A fluidity assay of potassium ion in *EdVP1* transgenic plant roots showed that potassium ion influx and H^+^ outflow in transgenic plants were higher than WT. Overexpressing *EdVP1* significantly improved yield and KUE of transgenic wheat and was related to higher K uptake capacity in root.

## Introduction

Potassium is an essential nutrient element for plants and is involved in osmoregulation and cell extension, stomatal regulation, activation of enzymes, photosynthesis, phloem loading, and transport of assimilates and water^1^, thereby promoting crop yield and quality. K deficiency in large areas of arable land worldwide limits sustainable development of agriculture, and threatens global food security^2^. In China, available K is relatively low in the soil, and with the extensive use of chemical fertilizer, K use efficiency is constantly decreasing, and consequently increasing the potential risk of environmental pollution and economic loss^3^.

Wheat is a main grain crop, with a planting area, total output, and total trade volume that rank first of all types of crops around the world. At present, with the increase of nitrogen and phosphorus fertilizer usage, lack of K fertilizer due to unbalanced fertilization in soil has become one of the main factors limiting wheat production in China^4–6^. Therefore, improving the use efficiency of K fertilizer is the only way to further increase wheat yield and save K fertilizer. At present, some genes related to K transport, metabolism, and regulation in plants have been reported. When the external concentration of K ions is lower than 100 μM, K ion transporter genes including *AtAKT1* and *AtHAK5* are essential in K uptake^7^. Activation of *AKT1* in *Arabidopsis* depends on CBL1 (calcineurin B-like 1) or CBL9-CIPK23 (CBL-interacting protein kinase) protein complexes^8^. Transcription factor *AtRAP2.11* also participates in the transcriptional regulation of *HAK5*^9^ in response to low K conditions. Overexpression of these genes can improve the ability of transgenic plants to tolerate low K stress. However, most transgenic plants have shown tolerance to low K stress only in greenhouses, with few reports showing transgenic plant tolerance to low K stress in the field, a necessary result for practical application value.

In plants, cytosolic inorganic pyrophosphate (PPi) is hydrolyzed by energy-conserving vacuolar-type H^+^-pyrophosphatases (V-PPases) that harness the free energy of PPi hydrolysis to establish transmembrane H^+^ gradients^10^; PPi also as an important integrator of metabolism and stress tolerance^11^. There are two phylogenetically distinct types of H^+^-pyrophosphatases (H^+^-PPases): type I and type II. The activity of the type I H^+^-PPase is dependent on cytosolic K^+^, and the type I H^+^-PPase is predominantly localized to the vacuolar membrane. In contrast, the activity of the type II H^+^-PPase is independent of K^+^, and is localized to the Golgi apparatus and related vesicles^12^. Up-regulation of either the *Arabidopsis* or *Thellungiella halophila* type I H^+^-PPase enhances growth/biomass and photosynthetic capacity in a variety of agriculturally important crops (such as *Arabidopsis*, maize, creeping bentgrass, and cotton) grown under normal or stressful conditions such as water scarcity and salinity stress^13–17^.

AVP1 (type I H^+^-PPase gene from *Arabidopsis*) is also believed to contribute to the establishment of electrochemical potential across the vacuole membrane, which is important for subsequent vacuolar secondary transport and ion sequestration^18^. Overexpression of *AVP1* also enhanced phosphorus nutrition in monocots and dicots^19^ and improved nitrogen use efficiency in romaine lettuce^20^. *AVP1* also contributes to high-Mg^2+^ tolerance in *Arabidopsis*^21^. Under normal or low phosphorus stress, the K content in the *AVP1* transgenic plant root system increased significantly, and was twice as much as the control plants^19,22^. H^+^-PPase serves as a multi-functional protein involved a variety of physiological processes in plants, some of which are not fully understood^21^. For instance, we still do not know whether the H^+^-PPase gene can affect the plant’s capacity to absorb K ions in the field under low K stress conditions.

We isolated the type I H^+^-PPase gene *EdVP1* from *Elymus dahuricus* (Gramineae), which has remarkable resistance to various abiotic stresses. Our previous research showed that overexpression of *EdVP1* in tobacco significantly improved the ability of transgenic plants to resist drought and high salt stresses^23^. In this study, *EdVP1* genes were transformed into winter wheat variety Zhengmai147 from north China and spring wheat variety Yangmai12 from south China and *EdVP1* gene functions were identified in soil from north and south China with low K content. After three consecutive years of a field experiment, the results showed that under different low K soils, overexpression of *EdVP1* significantly enhanced the uptake of K ions and increased grain yield in transgenic wheat compared to wild-type (WT) wheat. At the same time, analysis of the physiological mechanism proved that overexpression of *EdVP1* promotes K^+^ influx and H^+^ efflux in transgenic plant roots. These results show that expression of *EdVP1* can promote plant uptake of K and increase grain yield under low K stress, which indicates that *EdVP1* has important application prospects for genetic improvement of K use efficiency (KUE) in crops.

## Materials and Methods

### Plant materials

*Elymus dahuricus* was used to analyze the expression pattern of *EdVP1*. The wheat (*Triticum aestivum*) varieties Zhengmai147 and Yangmai12 were used to generate the transgenic wheat lines. Tobacco variety W38 was used to generate transgenic tobacco lines.

### Gene expression analyses following K treatments in *Elymus dahuricus*

Seedlings (7 d after germination) were grown for 21 d in a nutrient solution, and then transferred to a low potassium solution that contained 0.1 mM KNO_3_ as the sole K source for K induction. After the plants were treated with K for different durations, the leaves were collected for gene expression analysis. The plants were grown in plastic boxes that contained 10 L of nutrient solution; this solution was refreshed every 2 d. The nutrient solution for the hydroponic culture experiment was described previously^24^. The plastic boxes were placed in a growth chamber with a 16/8-h light/dark cycle.

### RT-PCR and northern blot

Total RNAs were extracted from plant tissues using a Trizol Kit (TaKaRa) and cDNAs were synthesized using the PrimeScript First-Strand cDNA Synthesis kit (TaKaRa cDNA Synthesis Kit, Dalian, Liaoning province, China) according to the manufacturer’s protocol. The primers used for RT-PCR were *EdVP1*-F (5′-GCTATTGCAAGTAGTCTCCGATTAGG-3′) and *EdVP1*-F (5′-CATCATGATTCTGTCTGCTCCATGCTC-3′); *Edactin*-F (5′-CAGTGGAGGTTCTACCATGTTTCC-3′) and *Edactin*-R (5′-CATGCAAGGCCATGCCATTGTG-3′). Total RNA (2 μg) was used for northern blot analysis; hybridization probes were produced by PCR as described above. A lane of driver (control) and one of tester (heat stress) were used for electrophoresis on a 1.0% formaldehyde agarose gel; the samples were then transferred onto a nylon membrane using iBlotter (Invitrogen, UK). Experimental procedures were performed as previously described^25^.

### Subcellular location of *EdVP1*

For subcellular location assays, *EdVP1* was inserted into the vector p16318 to fuse with green fluorescent protein (GFP) and produce the vector p16318-EdVP1, which was then biologically transformed into onion epidermal cells. The empty p16318:GFP vector was used as the control. The onion epidermis was placed in a dark environment for 16–24 h before treatment with a 2 M sucrose solution. Results were observed using a confocal laser scanning microscope (ZEISS LSM 700; Germany).

### Genetic transformation of wheat and tobacco

*EdVP1* was inserted into the plasmid pAHC25 under the control of the corn ubiquitin (Ubi) promoter (Fig. S1). Transgenic wheat plants were generated using the particle bombardment method^26^. Positive lines were detected by PCR using primers *Ubi*-F (5′-GCTCACCCTGTTGTTTGGTG-3′) and VP1-R (5′-TAAATCCCACCGCCTACACG-3′) in wheat. RT-PCR was conducted using primers actin-F (5′-CTCCCTCACAACAACCGC-3′) and actin-R (5′-TACCAGGAACTTCCATACCAAC-3′). To generate transgenic *EdVP1* tobacco (*Nicotiana tabacum*) plants, EdVP1 was inserted into the pBI121 vector under control of the CaMV 35 S promoter (Fig. S2), and constructs were transformed into wild-type W38 using *Agrobacterium*-mediated leaf disk transformation^27^. Positive transgenic EdVP1 tobacco were detected by PCR using primers F (5′-CGACTTCTTTGAGGTGAAGGAGGTAG-3′) and R (5′-CCAATTGCAAAACCCTTTCCGATGGC-3′). All T3 generation transgenic plants were used for analyses.

### Transgene copy number measurement in wheat by droplet digital PCR (dPCR)

We used the droplet digital PCR-based method for transgene copy number measurement in transgenic wheat as previously described^28^. The wheat PINb (*PUROINDOLINE*-b) gene as a single copy (homozygous) reference gene (two copies per hexaploid genome) has been previously employed to estimate transgene copy number. Reference genes were chosen in dPCR using primers PINb-D1b-F (5′-AGTTGGCGGCTGGTACAATG-3′) and PINb-D1b-R (5′-ACATCGCTCCATCACGTAATCC-3′). Primers and probe (PINb-D1b-P FAM-TCTCAACAATGTCCGCAGGAGCGGCC-BHQ1) were designed following the criteria specified by the instrument manufacturer. dPCR primer pairs and probe sequences were designed for *EdVP1* in wheat EdVP1-F (5′-ATCTACACTAAGGCTGCTGA-3′), EdVP1-R (5′-CTGGTTCAATCTCCTTGACA-3′), and probe EdVP1-P (HEX-CgCTgCTCTTgTTgTTgCCTCgATCTC-BHQ1). The samples were then transferred to a QX200 droplet reader (Bio-Rad). Droplet counts were analyzed, and transgene copy number measurements were generated using the Bio-Rad QuantaSoft™ software (V1.3.2) with default settings for threshold determination to distinguish positive and negative droplets.

### Field experiment

Field experiments were conducted from 2011 to 2014 at the Zhengzhou Fluvo-aquic Soil Fertility and Fertilizer Efficiency Long-Term Monitoring Base, which is part of the Institute of Wheat, Henan Academy of Agricultural Sciences, Henan province. Three consecutive field experiments were conducted in the 2011–2012 (T4 generation), 2012–2013 (T5 generation), and 2013–2014 (T6 generation) growing seasons. All experiments had two treatments each with four replications. K concentration was determined as previously described^29^.

In 2011–2012, both treatments were conducted as a plot trial (Fig. S11). The normal K treatment had 14.0 g K/m^2^ in the form of potassium chloride applied prior to sowing, and the low K treatment had no K application. In both treatments, 18.7 g/m^2^ phosphorus (P) was applied as calcium superphosphate and 33.3 g/m^2^ nitrogen (N) was applied as urea. Each genotype was planted in soil-filled plots in each replicate; plots were 1.5 m^2^, which included seven rows spaced 20 cm apart (Fig. S11). The sowing density was set at 22 germinating seeds per row. At maturity, ten plants in each plot were randomly collected to measure grain weight per plant, grain number, 1000-grain weight (TGW), tiller number, and plant height. Five plants in each plot were randomly collected to measure K concentration in straw and grain.

In 2012–2013, both treatments were conducted as a field trial (Fig. S11). The normal K treatment had 17.3 g K/m^2^ in the form of potassium chloride applied prior to sowing, and the low K treatment had 13.8 g K/m^2^ application. In both treatments 68.5 g/m^2^ phosphorus (P) was applied as calcium superphosphate and 36.6 g/m^2^ nitrogen (N) was applied as urea. Each genotype was planted in the field with three replicates; each plot was 48 m^2^, which included three rows spaced 20 cm apart. The sowing density was set at 270 germinating seeds per m^2^. At maturity, actual grain yield was recorded in the whole plot. Twenty plants in each plot were randomly collected to measure K concentration, grain weight per plant, grain number per spike, TGW, dry weight of shoot, tiller number, plant height, and panicle length. K utilization efficiency (KUE) at maturity was estimated as grain yield/potassium chloride applied. The nutritional composition of the soils before wheat was planted in each field are summarized in Table S6.

In 2013–2014, both treatments were conducted at another pilot site (Fig. S11), which was also part of the Fertilizer Efficiency Long-Term Monitoring Base, where basic fertilizer was deficient in potassium. Low potassium treatment had 4.5 g K/m^2^ application in the form of potassium chloride, 22.4 g/m^2^ phosphorus (P) as calcium superphosphate, and 30.0 g/m^2^ nitrogen (N) as urea applied prior to sowing. The normal K treatment had 7.5 g K/m^2^ in the form of potassium chloride, 37.5 g/m^2^ phosphorus (P) as calcium superphosphate, and 30.0 g/m^2^ nitrogen (N) as urea applied prior to sowing. Each plot was 667 m^2^, grain yield was recorded in the whole plot, and spike densities were recorded on two rows that were each 1 m long in each plot. The nutritional composition of the soils before wheat was planted in each field are summarized in Table S7.

### Pot experiment

The pots used had a diameter of 10 cm and height of 20 cm. Each pot contained 0.5 kg soil. Soil samples (0–20 cm depth) used in the pot experiment were red soil and black soil, which were obtained from Zhengzhou, Henan Province and Jiujiang, Jiangxi Province, respectively. The content of potassium in the soil retrieved from the field was low (Table S8). We applied different levels of potassium fertilizer to treat wheat and analyze the differences in potassium utilization between transgenic wheat and WT. In addition, according to the content of other elements including nitrogen and phosphorus in the soil, we supplemented fertilizer to make other elements sufficient. Here, there was only a difference in potassium fertilizer. K application was divided into four levels of K0 (0 g/m^2^), K1 (7.5 g/m^2^), K2 (15.0 g g/m^2^), and K3 (22.0 g/m^2^) in the form of potassium chloride. Each pot contained four seedlings and each treatment was replicated three times. The entire experiment was carried out in a greenhouse with a 16/8-h light/dark cycle. After 30 days, biomass and K content in each part of the plant and chlorophyll content were determined.

### Determining the absorption mechanism of K in transgenic tobacco

Seeds of wild type (W38) and transgenic tobacco were surface-sterilized and sown on MS medium. Seven-day-old seedlings grown under normal MS medium (1 mM potassium) were dipped into different media with 0.05 mM, 0.1 mM, and 0.6 mM K. After two weeks, K accumulation and chlorophyll content were determined. For low K stress resistance assays, stalks (three-week-old seedlings grown on MS medium) were cut into 1 cm pieces containing leaf buds and transferred to 0.05 mM K MS medium; phenotypes were observed four weeks later. For analysis of K absorption under low K stress, seven-day-old seedlings were transferred to Hoagland’s solution containing 0.05 mM K. After four weeks, root vitality and V-type H^+^-PPase were determined by measuring the release of inorganic phosphate (Pi) as described previously^30^, and free IAA content was determined^31^.

### Measurement of Net K^+^/H^+^ Flux Rate in Plants with NMT

Tobacco seedlings were grown in nutrient solution for three weeks and then deprived of 0.05 mM K for 7 d. Net K^+^ flux and H^+^ flux in the root apical zone, elongation zone, and root hair zone were measured using non-invasive micro-test technology (NMT) as described previously^32^. Wheat seedlings (7 days after germination) were grown for 7 days in a nutrient solution that contained 0.1 mM K^+^. The roots of these plants were then transferred to a measuring solution containing 0.1 mM KNO_3_, 0.1 mM CaCl_2_, and 0.3 mM MES (pH6.0), and allowed to balance for 10 min. The net K^+^ fluxes were measured as described previously^33^. The maximum K^+^ flux rates along the root were recorded.

### Growth of *VP1* mutant under different K treatments

Seeds of wild type (Col-0, WT) and *VP1* mutant were surface-sterilized and sown on MS medium. Materials were placed in a greenhouse with a photoperiod of 16 h light/8 h darkness. Seven-day-old seedlings grown on MS medium were transferred to different MS medium containing 50 µM K, 10 µM K, and 0 µM K. MS medium was used as the control with 1 mM K. After ten days of growth, roots were systematically analyzed using a WinRHIZO Pro 2012 (EPSON Flatbed Scanner EPSON Expression 10000XL 1.8 V3.45 3.4)

### Statistical analysis

One-way analysis of variance (ANOVA) was performed with SPSS12.0 for Windows (SPSS Inc., Chicago, Illinois, US)

## Results

### The expression pattern, phylogenetic relationships, and subcellular localization of *EdVP1*

**Figure 1:**
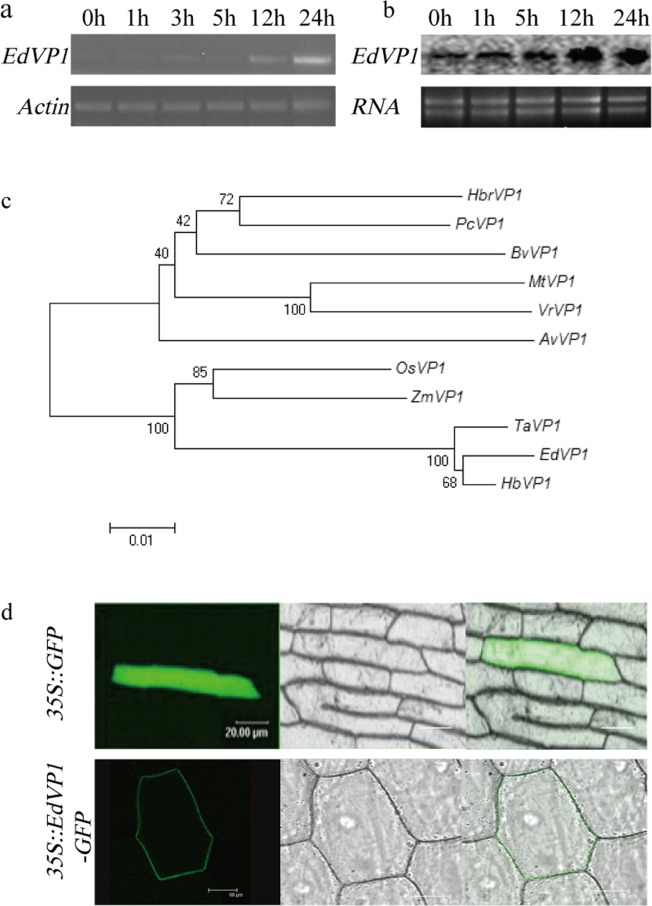
The expression pattern of *EdVP1*, homology analyses, and subcellular localization. (**a**) RT-PCR analysis of expression level of EdHP1 under low K treatment. (**b**) Northern blot analysis of expression level of EdHP1 under low K treatment (**c**) Homology analyses of amino acid sequence of EdHP1 and H^+^-PPase from other plant species using DNAMAN software. The accession numbers of all genes are as follows: *Hordeum brevisubulatum*, *HbVP1* (AY255181); *Oryza sativa*, *OsVP1* (AB012765); *Zea mays*, *ZmVP1* (YP_002219); *Arabidopsis thaliana*, *AVP1* (M81892); *Beta vulgaris*, *BvVP1* (AAA61609); *Hevea brasiliensis*, *HbrVP1* (AB032839); *Pyrus communis*, *PcVP1* (NM_101437); *Medicago truncatula*, *MtVP1* (AAS70856); *Vigna radiate*, *VrVP1* (VRU31467); and *Triticum aestivum*, *TaVP1* (L32791). (**d**) Subcellular localization of EdHP1 in onion epidermal cells.

The results of RT-PCR and northern blot showed that the expression level of *EdVP1* increased under low potassium stress treatment (0.1 mM potassium) and peaked at 24 h after treatment (Fig. 1a,b). The evolutionary relationships of *EdVP1* homologues from different plant species are shown in Fig. 1c. Results indicated that the amino acid sequence of the H^+^-PPase gene from different plant species is conserved, and the *EdVP1* gene has higher homology with homologous genes from wheat (*TaVP1*) and *Hordeum brevisubulatum* (*HbVP1*). The results of subcellular localization showed that *EdVP1*-*GFP* fusion protein was localized to the plasma membrane, and control GFP was distributed throughout the cell (Fig. 1d), which indicated that EdVP1 is V-type H^+^-PPase localized in the plasma membrane.

### Overexpression of *EdVP1* increases grain yield and K utilization efficiency in the field

**Figure 2:**
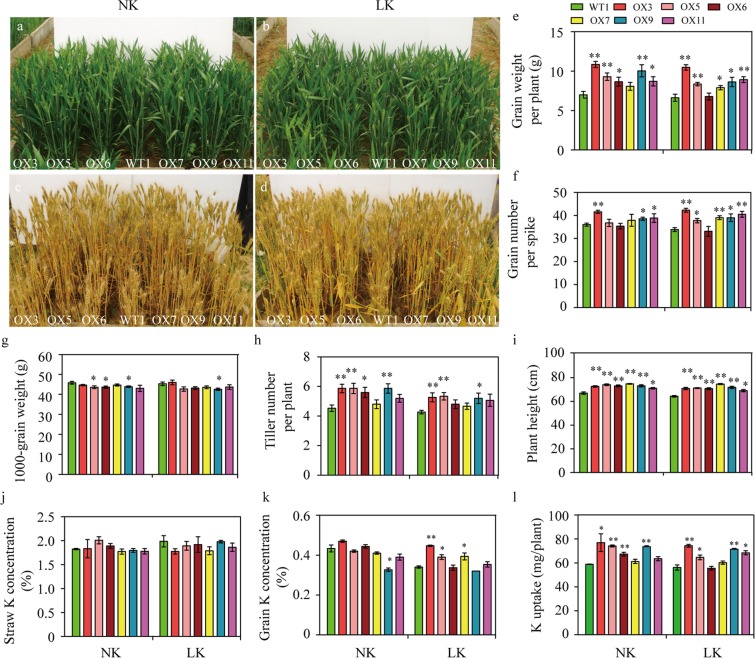
*EdVP1* transgenic wheat exhibited improvements in K uptake and yield performance in a plot trial. (**a**,**c**) Performance of transgenic lines and the wild type Zhengmai147 under normal K conditions (NK) in the heading period (**a**) and mature stage (**c**). (**b**,**d**) Performance of transgenic lines and the wild type Zhengmai147 under low K conditions (LK) in the heading period (**b**) and mature stage (**d**). (**e**) Grain weight per plant (**g**). (**f**) Grain number per plant. (**g**) 1000-grain weight (TGW) (**g**). (**h**) Tiller number per plant. (**i**) Plant height (cm). (**j**) Straw K concentration (%). (**k**) Grain K concentration (%). (**l**) K uptake (mg/plant). Data represented as means ± SE of three replicates. * and ** Indicate that differences between the wild type and the transgenic lines were significant at the P < 0.05 and 0.01 level, respectively.

To explore the biological functions of *EdVP1*, we overexpressed this gene in the winter wheat accession Zhengmai147 (wild-type plants, WT1), and obtained six independent transgenic lines through PCR assay (Fig. S3a). Using semi-quantitative RT-PCR (Fig. S3b), we confirmed the higher expression level of *EdVP1* in four independent T4 overexpression lines compared to WT1 (Fig. S3). We selected line 5 and line 9, which had a high expression level of *EdVP1* in transgenic wheat, and analyzed the copy number by dPCR. The results showed that the transgenic lines (OX5 and OX9) had four copies and one copy, respectively (Fig. S4). Next we preliminarily evaluated and selected for the in-field performance of the overexpression lines under low K conditions (no K application) in the 2011–2012 growing season. At heading and maturity stages, phenotypes including plant height and biomass of transgenic wheat increased significantly compared with WT1 under normal (14.0 g K/m^2^ application) and low potassium treatments (Fig. 2a–d). The grain yield per plant of four transgenic wheat lines (OX3, OX5, OX9, and OX11) significantly increased compared to WT1 under both normal K (NK) and Low K (LK) conditions (P < 0.05) (Fig. 2e and Table S1).

We further analyzed other agronomic traits of *EdVP1* transgenic wheat. Three transgenic lines, OX3, OX5, and OX9, had higher grain number per spike and tiller number per plant under low K (P < 0.05) (Fig. 2f,h and Table S1). The 1000-grain weights (TGWs) of two transgenic wheat lines (OX5 and OX6) were significantly reduced compared to WT1 under normal K conditions; only line OX9 had significantly lower TGW than WT1 under low K conditions (Fig. 2g and Table S1). Those results suggested that increased yield of transgenic wheat under low K is mainly due to the increase of grain number per spike and tiller number per plant. In addition, the plant height of WT1 plants was significantly shorter than that of transgenic lines under normal K or low K conditions (Fig. 2i) (P < 0.01).

We also investigated K concentration in straw and grain and found that grain K concentration in only three transgenic wheat lines (OX3, OX5, and OX7) was higher than WT1 (Fig. 2j,k) (P < 0.05). Under both LK and NK conditions, the K uptake per plant of three transgenic lines (OX3, OX5, and OX9) was significantly higher than WT1 (Fig. 2l) (P < 0.05). Based on results of the grain yield and K uptake, the three transgenic lines OX3, OX5, and OX9 were selected for further study.

**Figure 3:**
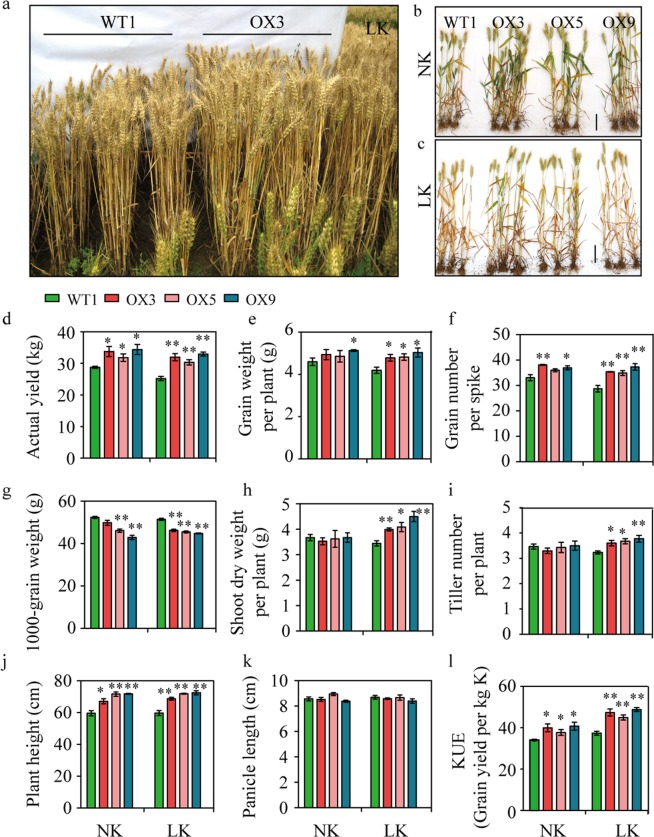
Overexpression of *EdVP1* in wheat increases grain yield and K utilization efficiency in the field. (**a**) Field performance of transgenic lines and the wild type Zhengmai147 under LK conditions. (**b**) Plant performance under NK. (**c**) Plant performance under LK. (**d**) Actual yield (kg) under NK and LK. (**e**) Grain weight per plant (**g**). (**f**) Grain number per spike. (**g**) 1000-grain weight (TGW) **(g**). Shoot dry weight per plant (**g**). (**i**) Tiller number per plant. (**j**) Plant height (cm). (**k**) Panicle length (cm). (**l**). K utilization efficiency. Data represented as means ± SE of three replicates. * and ** Indicate that differences between the wild type and the transgenic lines were significant at the P < 0.05 and 0.01 level, respectively.

In 2012–2013, we expanded the planting area of transgenic wheat. The plant height and biomass of transgenic wheat under low K conditions were significantly higher than those of WT1, which was consistent with the results of the previous year (Figs. 3a–c). The grain yield of three transgenic lines (OX3, OX5, and OX9) increased 20.29–30.58% and 10.75–19.68% compared to WT1 under low K (P < 0.01) and normal K treatment conditions (P < 0.05), respectively (Fig. 3d, Table S2). We found that the grain weights per plant of all transgenic wheat lines were significantly higher than those of WT1 (P < 0.05) only under low K conditions (Fig. 3e, Table S2).

We analyzed the three major yield components (the number of grains per spike, tiller number per plant, and TWG) and found that compared with results from the previous year, the number of grains per spike of transgenic wheat were still significantly increased under normal K and low K conditions compared with WT1 (P < 0.05) (Fig. 3f, Table S2). The number of tillers per plant of all transgenic wheat lines were significantly increased compared to WT1 under low K conditions (P < 0.05) (Fig. 3i, Table S3). The TGW of transgenic wheat was lower than that of WT1 (Fig. 3g, Table S2). The plant height of transgenic wheat was significantly higher than that of WT1, which was similar with results of the previous year (P < 0.01) (Fig. 3j, Tables S1 and S3). The shoot dry weight per plant in transgenic wheat under low K was significantly higher than that of WT1 (P < 0.05) (Fig. 3h, Table S3). There was no significant difference in spike length between WT1 and transgenic wheat. Compared with WT1 plants, the KUE (defined as the grain yield per unit of available K in the soil) increased by 20.29% to 30.58% and 10.75% to 19.68% in *EdVP1* transgenic plants under the low and normal K conditions, respectively (Fig. 3l).

In the 2013–2014 growing season, we further increased the area of field experimental plots to analyze yield changes of transgenic wheat (lines OX5 and OX9) under low fertilizer conditions. The results indicated that actual grain yield of OX5 and OX9 increased 7.14–10.79% compared to WT1 under low fertilizer conditions (Fig. S5). The results of yield components showed that the two transgenic lines had higher grain number per spike under both low and normal fertilizer conditions, and TGW and number of tillers per square meter were not different between transgenic wheat and WT1 (Fig. S5), which was similar to results of the previous two years.

### *EdVP1* transgenic wheat had higher biomass and K uptake under K treatment conditions

**Figure 4:**
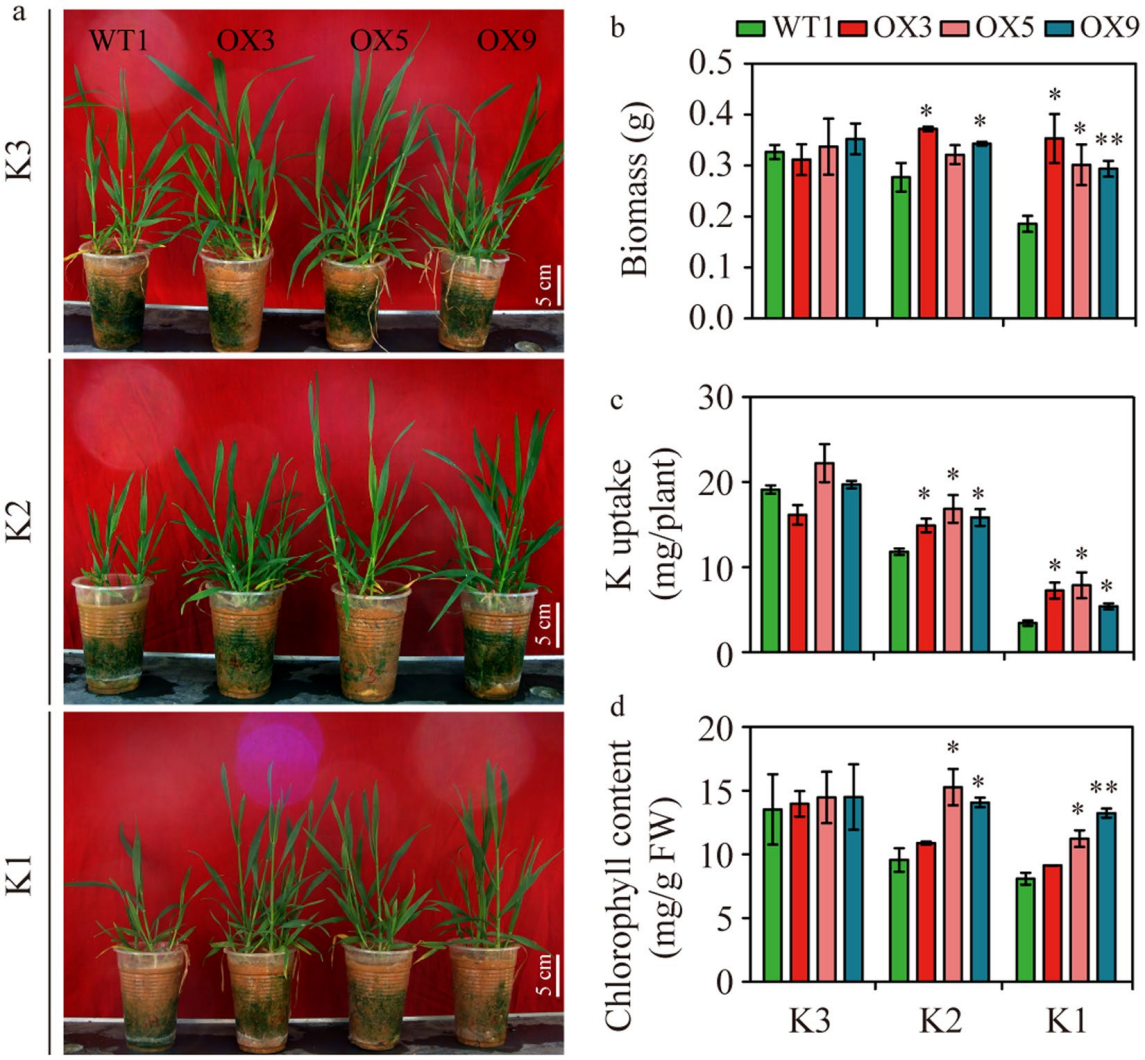
*EdVP1* overexpression in wheat increased biomass and K uptake in wheat seedlings. (**a**) Performance of transgenic lines and the wild type Zhengmai147 under different K treatments in red soil (K1, K2, and K3). K1, low potassium level; K2, medium potassium level; K3, high potassium level. (**b**) Biomass per plant (**g**). (**c**) K uptake per plant. (**d**) Chlorophyll content. Data represented as means ± SE of three replicates. * and ** Indicate that differences between the wild type and the transgenic lines were significant at the P < 0.05 and 0.01 levels, respectively.

We carried out a pot experiment at the seedling stage to analyze *EdVP1* functions under different K treatment conditions. We compared the performance of WT1 (Zhengmai147) and the transgenic lines (OX3, OX5, and OX9) in pots with red soil (K deficient soils from southern China) under three levels of K treatment (K1 7.5 g/m^2^, K2 15.0 g/m^2^, and K3 22.0 g/m^2^). For the WT1 plants, both low-K treatments (K1 and K2) significantly reduced biomass per plant as compared with the high-K treatment (K3), but transgenic wheat had only slight changes under different K treatment conditions (Fig. 4a,b). Compared with WT1, all three transgenic lines had significantly higher biomass per plant and K uptake per plant under K1 and K2 conditions (Fig. 4b,c). Results of the chlorophyll content assay showed that chlorophyll content of the transgenic lines OX5 and OX9 was significantly higher than WT1 under K1 and K2 conditions (Fig. 4d).

In another pot trial with black soil (K deficient soils from northern China) under four levels of K treatment (K0, K1, K2, and K3), we compared the performance of WT1 (Zhengmai147) and the transgenic lines (OX3, OX5, and OX9). All three transgenic lines had significantly higher biomass per plant, K uptake per plant, and chlorophyll content under no K or low K conditions (K0, K1, and K2) (Fig. S6). We also completed a pot trial in the same two soils for transgenic wheat of variety Yangmai12. Most transgenic lines also had significantly higher biomass per plant and uptake per plant under low K conditions (Table S4), which indicated that overexpression of *EdVP1* in different wheat varieties confers higher biomass and K uptake in different levels of low K soil compared to WT.

### Overexpression of *EdVP1* increases potassium influx in transgenic plant root

To further explore the molecular mechanism of increased K uptake observed in *EdVP1* transgenic wheat, *EdVP1* was initially overexpressed in tobacco, and the dynamic process of K^+^ flow in transgenic tobacco roots was monitored using non-invasive micro-test technology (NMT). Semi-quantitative RT-PCR showed that *EdVP1* was effectively transcribed in these T4 generation tobacco lines, and the highest expression of line #32 was selected to explore the low potassium treatment concentration. Seven-day-old seedlings grown under normal MS medium (1 mM potassium) were not significant difference between transgenic and WT plants, which were dipped into different media with 0.05 mM, 0.1 mM, and 0.6 mM K, and after 14 days, we found that the *EdVP1* overexpression tobacco plants (#32) had higher K accumulation and chlorophyll content than WT under low K conditions (MS containing 0.05 mM potassium) (Fig. S7). No significant phenotypic differences were observed between transgenic and WT plants under high K conditions (MS containing 0.1 and 0.6 mM potassium). We then moved the stem segments of tobacco into the low K medium (MS containing 0.05 mM potassium). After four weeks of treatment, compared with WT, all three transgenic lines (#11, #12, and #32) had significantly longer shoots and length of total roots, significantly higher K accumulation in shoot and root, and H^+^-PPase activity in shoots was higher than in WT plants under low K conditions. The WT shoot did not root on low K medium (Fig. S8).

**Figure 5:**
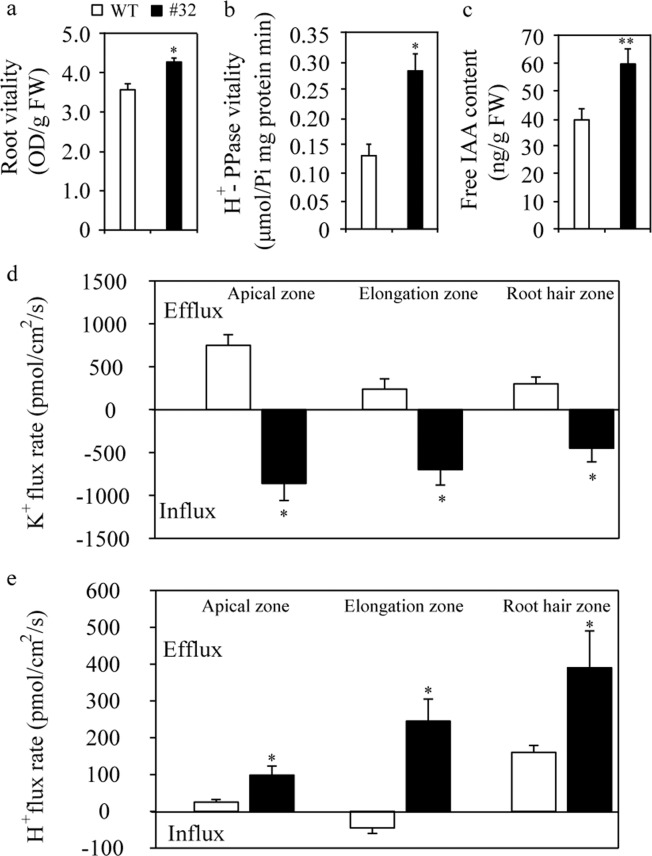
Overexpression of *EdVP1* increases potassium influx in transgenic tobacco. (**a**) Root vitality under low K conditions. (**b**) H^+^-PPase vitality under low K conditions. (**c**) Free IAA content under low K conditions. (**d**) K^+^ flux rate at the root apical zone, elongation zone, and root hair zone. (**e**) H^+^ flux rate at the root apical zone, elongation zone, and root hair zone. Data are represented as means ± SE of three replicates. * and ** Indicate that differences between the wild type and the transgenic lines were significant at the P < 0.05 and 0.01 levels, respectively.

To further determine whether transgenic *EdVP1* plants also display improved tolerance to long-term K starvation, seven-day-old seedlings grown under normal conditions were dipped into Hoagland’s solution containing 0.05 mM potassium. We found that the transgenic plants had longer main stems, and higher root number, root surface areas, and root volume than WT under low K conditions (Table S5). We also compared root vitality, H^+^-PPase activity, and free plant hormone-indole-3-acetic acid (IAA) content between the transgenic and WT plants, and found that all three were significantly higher in transgenic line #32 than WT plants under low K conditions (Fig. 5).

We further measured the K^+^ fluxes at the root surface using NMT. The results showed that transgenic lines had higher K^+^ influx rates and H^+^ efflux rates than WT under low K treatment (Fig. 5). Moreover, the VP1 gene mutant in *Arabidopsis* (*avp1*) showed obvious phenotypic differences from WT like shorter total root length, and reduced numbers of lateral and hairy roots and root surface area compared with those of the WT under low K conditions (Fig. S9). To understand the mechanisms by which EdVP1 increased N uptake in wheat, we further measured the K^+^ fluxes at the root surface using transgenic wheat. The three transgenic lines (OX3, OX5, and OX9) displayed significantly higher K^+^ influx rates than WT1 plants (Zhengmai147) in a measuring solution containing 0.1 mM K for 10 min (Fig. S10). These results indicate that overexpressing *EdVP1* increased root length and root number, and improved root vitality under low K conditions by enhancing K^+^ influx rates in plants.

## Discussion

### *EdVP1* can increase the yield of transgenic wheat by increasing the number of grains per spike under low potassium conditions

Field experiments for three consecutive years showed that the yield of *EdVP1* transgenic wheat was significantly increased under low potassium conditions compared with WT (Figs. 2 and 3 and S2; Table S1). At the same time, the results of yield component analysis showed that in the first year of the field experiment, the number of grains per spike and spike number of transgenic wheat increased significantly compared with WT, but the 1000-grain weight (TGW) significantly decreased (Figs. 2 and 3, S2). In the field experiments in the following year, the experimental area gradually increased. The results showed that only the number of grains per spike of transgenic wheat increased, while the number of spikes and the TGW did not change significantly (Fig. S5). This change may be due to the increase of planting area in the field, leading to increased plant density, which limited increases of spike number of transgenic wheat, while the number of grains per spike still increased. Therefore, multiple years of field experiments have shown that overexpression of *EdVP1* increased the yield of transgenic wheat under low K conditions due to the increase of grain number per spike.

K can increase the root growth and vegetative mass of cereals, which results in increased photosynthetic capacity, particularly during dry seasons. The availability of assimilates often determines the number of aborted grains in cereals^34^. In this study, we found that in pot experiments, K uptake in transgenic wheat was significantly improved and per plant biomass increased under low K conditions (Figs. 4, S3). At the same time, overexpression of *EdVP1* in tobacco promoted root growth and led to higher K accumulation in shoot and roots (Fig. S8 and Table S5), and plants with the VP1 gene mutant in *Arabidopsis*, *avp1*, had a weak root system compared to WT (Fig. S9). These results indicated that *EdVP1* transgenic plants can uptake more K and have more developed roots, which are related to the increase of grain number per spike and yield of transgenic wheat.

We transformed *EdVP1* into two wheat varieties that are suitable for planting in southern and northern China. We completed consecutive yield experiments in the field under low K conditions in Zhengzhou, Henan Province. The results showed that the yield of transgenic wheat was higher than that of WT in experimental locations (Figs. 2 and 3, and S2, Table S2). At the same time, two kinds of low K soils, red soil and black soil from southern and northern China, respectively, were selected for pot experiments. The results showed that the biomass and K uptake capacity of transgenic wheat under low K conditions were significantly higher than that of WT (Fig. S7 and S3, Table S4). These results indicated that *EdVP1* can function in different wheat receptor backgrounds and in different low K soil environments, which proves that *EdVP1* has important application value in saving K fertilizer and promoting K use efficiency (KUE) in wheat.

### *EdVP1* might promote K^+^ uptake by affecting the distribution of auxin in roots and the activity of K^+^ transporter in the plasma membrane

We showed that *EdVP1* can improve K uptake and KUE of transgenic wheat. H^+^-PPase results in an acidic environment in plant roots, which is beneficial to the distribution of auxin IAA and further promotes the development of plant roots^35^. In this study, we observed the root phenotype of *EdVP1* transgenic tobacco and *AVP1 Arabidopsis* mutant under low potassium conditions. The results showed that *EdVP1* promoted the development of plant roots (Fig. S9), which increased root surface area (Table S5), thereby promoting K uptake. On the other hand, we found that the inflow rate of K^+^ in the roots of *EdVP1* transgenic plants (tobacco and wheat) was higher than that of WT, and the efflux rate of H^+^ was higher than that of WT. Subcellular localization analysis showed that *EdVP1* was located on the plasma membrane.

Most *VP1*-like proteins are localized on the vacuole membrane, which can promote the vacuole to regulate ion balance in the cytoplasm. VP1 localized on the plasma membrane may affect plant vascular tissue cells and affect ion transport through vascular tissue^36^. Therefore, we suggest that *EdVP1* might form a proton gradient between the inside and outside of the plasma membrane, which can drive the K^+^ transporter on the plasma membrane to transport K^+^ inside^37^, resulting in higher K^+^ uptake rate in *EdVP1* transgenic plants. This proton gradient can also affect the activity of other ion transporters on the plasma membrane, thereby improving the resistance of *VP1*-like transgenic plants to various abiotic stresses, including low phosphorus, high salinity^36^, and drought^17,38^. On the other hand, the efflux of H^+^ might promote secretion of organic acids in the rhizosphere of plants, which can facilitate K^+^ mineralization and further promote the absorption of more K^+^ in the root of plants^39,40^. In short, these two mechanisms work together to promote the uptake of more K in *EdVP1* transgenic plants and improve KUE.

## Supplementary information

Supplementary information.
