# Supplementary material for: Overexpression of V-type H+ pyrophosphatase gene EdVP1 from Elymus dahuricus increases yield and potassium uptake of transgenic wheat under low potassium conditions
Source: Sci Rep. 2020 Mar 19;10:5020. doi: 10.1038/s41598-020-62052-5 (PMC7081212; doi:10.1038/s41598-020-62052-5)
Supplement: Supplementary file 1 — Supplementary information. [file 41598_2020_62052_MOESM1_ESM.pdf]

Overexpression of V-type H^+ pyrophosphatase gene *EdVP1* from *Elymus dahuricus* increases yield and potassium uptake of transgenic wheat under low potassium conditions

Yongbin Zhou[1, #], Yan Li[2, #], Xueli Qi[2], Rongbang Liu[1], Jianhui Dong[1], Weihuan Jing[1], Mengmeng Guo[1], Qinglin Si[1], Zhaoshi Xu[1], Liancheng Li[1], Chengshe Wang[3], Xianguo Cheng[4, †], Youzhi Ma[1, †], Ming Chen[1, †]

Supplementary materials

Table S1. Agronomic traits of *EdVP1* transgenic wheat in field experiments under normal and low potassium conditions (Zhengzhou, 2012)
Table S2. Actual yield of *EdVP1* transgenic wheat in field experiments under normal and low potassium conditions (Zhengzhou, 2013)
Table S3. Agronomic traits of *EdVP1* transgenic wheat in field experiment under normal and low potassium conditions (Zhengzhou, 2013)
Table S4. Performance of the wild type (Yangmai12) and transgenic lines in pot trials of two soils under different K treatment
Table S5. Transgenic tobacco root morphological parameters under treatment with 0.05 mmol L^{-1} K
Table S6. Soil environment at different field trial locations in 2012 in Zhengzhou
Table S7. Soil environment at different field trial locations in 2013 in Zhengzhou
Table S8. Soil environment at different pot trial locations

Fig. S1. The map of expression vector of EdVP1 in transgenic wheat
Fig. S2. The map of expression vector of EdVP1 in transgenic tobacco
Fig. S3. PCR detection
Fig. S4. Droplet digital PCR analysis for determining copy number
Fig. S5. *EdVP1* transgenic wheat exhibited improvements in a variety of yield performance traits in the 2013-2014 growing season.
Fig. S6. *EdVP1* overexpression in wheat increased biomass and K uptake in wheat seedlings.
Fig. S7. *EdVP1* overexpression in tobacco plants showed different K accumulation and chlorophyll content in MS medium (containing 0.05 mM, 0.1 mM, and 0.6 mM K)
Fig. S8. Overexpression of *EdVP1* improved adaptation to potassium (K) starvation in transgenic tobacco plants.
Fig. S9. Phenotypes of the wild type and the *VP1* mutant after different K treatments
Fig. S10. K^+ flux rate at the root surface of *EdVP1* transgenic wheat.
Fig. S11. The plot and field trials were conducted from 2011 to 2014 at the Zhengzhou Fluvo-aquic Soil Fertility and Fertilizer Efficiency Long-Term Monitoring Base

Table S1. Agronomic traits of *EdVP1* transgenic wheat in field experiments under normal and low potassium conditions (Zhengzhou, 2012)

Normal K	Grain weight per plant (g)	Grain number per spike	1000-grain weight (g)	Tiller number per plant	Plant height (cm)
WT1	7.00±1.87	36.04±0.66	45.83±2.68	4.53±0.83	67.07±3.49
OX3	10.86±1.52**	41.59±0.66**	44.63±1.26	5.87±1.13**	72.67±2.58**
OX5	9.30±1.84**	36.78±1.56	43.58±2.70*	5.87±1.36**	74.13±2.75**
OX6	8.67±2.19*	35.35±1.21	43.66±2.06*	5.60±1.35*	73.13±2.90**
OX7	8.08±1.95	37.92±2.59	44.68±2.01	4.80±1.15	74.80±1.26**
OX9	10.05±2.99**	38.58±0.68*	43.87±1.37*	5.87±1.25**	73.20±3.51**
OX11	8.72±2.25*	38.90±0.35*	43.04±6.09	5.20±1.01	71.13±2.36*
Low K	Grain weight per plant	Grain number per spike	1000-grain weight (g)	Tiller number per plant	Plant height (cm)
WT1	6.63±1.74	33.90±0.84	45.30±3.42	4.27±0.46	64.33±2.55
OX3	10.48±1.52**	42.30±0.86**	45.99±4.42	5.27±1.16**	70.87±3.83**
OX5	8.36±0.95**	37.75±0.95*	42.68±4.24	5.33±0.98**	71.27±1.53**
OX6	6.78±1.92	33.14±2.14	43.18±2.75	4.80±1.15	70.80±2.93**
OX7	7.90±1.16*	39.08±0.84**	43.54±2.60	4.67±0.82	74.67±1.91**
OX9	8.63±2.56*	39.03±1.65*	42.57±2.16*	5.20±1.37*	71.87±3.52**
OX11	8.93±1.61**	40.54±1.27**	43.68±4.37	5.07±1.58	69.13±3.87*

Data represent means ± SE of three replicates. * and ** indicate that differences between the wild type and the transgenic lines were significant at the P < 0.05 and 0.01 levels, respectively. The field tests were completed in Zhengzhou with three replicates and at least five plants for each repeat were investigated.

Table S2. Actual yield of *EdVP1* transgenic wheat in field experiments under normal and low potassium conditions (Zhengzhou, 2013)

Normal K	Actual yield (kg)	YI(%)	Grain weight per plant (g)	Grain number per spike	1000-grain weight (g)
WT1	28.74±0.33		4.60±0.18	33.07±1.16	52.38±0.50
OX3	33.74±1.60*	17.39	4.94±0.24	38.13±0.18**	49.86±1.18
OX5	31.83±1.17*	10.75	4.86±0.27	36.03±0.60	46.11±0.79**
OX9	34.40±1.62*	19.68	5.13±0.04*	36.94±0.81*	42.95±1.01**
Low K	Actual yield (kg)	YI(%)	Grain weight per plant (g)	Grain number per spike	1000-grain weight (g)
WT1	25.23±0.64		4.19±0.15	28.75±1.26	51.42±0.50
OX3	31.98±1.15**	26.73	4.78±0.16*	35.38±0.10**	46.26±0.51**
OX5	30.35±0.84**	20.29	4.82±0.15*	34.93±0.93**	45.52±0.46**
OX9	32.95±0.64**	30.58	5.04±0.21*	37.33±1.29**	44.84±0.25**

Actual yield, yield of grains in plot (48 m^2). The field tests were completed in Zhengzhou with three replicates and at least twenty plants for each repeat were investigated. Data represent means ± SE of three replicates. * and ** indicate that differences between the wild type and the transgenic lines were significant at the P < 0.05

and 0.01 levels, respectively.

Table S3 Agronomic traits of *EdVP1* transgenic wheat in field experiments under normal and low potassium conditions (Zhengzhou, 2013)

Normal K	Shoot dry weight per plant (g)	Tiller number per plant	Plant height (cm)	Panicle length (cm)
WT1	3.67±0.12	3.47±0.10	59.62±1.59	8.56±0.16
OX3	3.53±0.14	3.30±0.11	67.19±1.60*	8.52±0.15
OX5	3.62±0.33	3.43±0.20	71.67±1.35**	8.95±0.12
OX9	3.67±0.18	3.50±0.18	71.80±0.26**	8.38±0.08
Low K	Shoot dry weight per plant (g)	Tiller number per plant	Plant height (cm)	Panicle length (cm)
WT1	3.45±0.10	3.23±0.06	59.75±1.52	8.69±0.15
OX3	3.99±0.06**	3.60±0.11*	68.74±0.87**	8.58±0.06
OX5	4.09±0.18*	3.68±0.10*	71.93±0.28**	8.67±0.21
OX9	4.50±0.20**	3.78±0.13**	72.54±1.30**	8.40±0.18

At least twenty plants for each repeat were investigated. Data represent means ± SE of three replicates. * and ** indicate that differences between the wild type and the transgenic lines were significant at the P < 0.05 and 0.01 levels, respectively.

Table S4 Performance of the wild type (Yangmai12) and transgenic lines in pot trials of two soils under different K treatments

	Lines	K uptake (mg/plant)				Biomass (g/plant)			
		K3	K2	K1	K0	K3	K2	K1	K0
Red soil	WT2	13.31±0.51	14.23±0.76	8.70±0.08	3.79±0.39	0.12±0.00	0.31±0.01	0.25±0.01	0.16±0.02
	Y121	16.90±0.33**	22.60±1.25**	10.79±0.27**	5.27±0.51	0.37±0.04**	0.41±0.03*	0.30±0.00*	0.15±0.01
	Y122	16.27±1.16*	21.22±1.80*	9.37±0.01**	4.72±0.21	0.34±0.01**	0.41±0.02*	0.24±0.00	0.15±0.04
	Y123	15.33±0.38*	20.22±1.08*	9.60±0.25*	1.93±0.29	0.36±0.01**	0.39±0.02*	0.26±0.03	0.16±0.04
	Y124	15.59±0.53*	27.76±0.46**	14.08±0.84**	3.86±1.39	0.31±0.02**	0.55±0.06*	0.30±0.01*	0.09±0.01
	Y125	12.91±0.91	19.64±2.87	8.84±0.16	2.50±0.28	0.28±0.01**	0.28±0.01	0.23±0.00	0.09±0.02
Black soil	WT2	16.35±1.05	14.98±1.02	15.31±0.22	11.29±0.04	0.57±0.00	0.53±0.01	0.52±0.01	0.49±0.02
	Y121	21.69±1.69*	18.65±0.79*	17.74±0.33**	15.67±0.35**	0.64±0.02*	0.58±0.00**	0.62±0.02*	0.56±0.01*
	Y122	20.75±0.81*	19.75±0.86*	18.17±0.30**	13.50±0.12**	0.63±0.01**	0.57±0.01*	0.52±0.06	0.58±0.01*
	Y123	17.27±1.41	17.43±0.09	17.30±1.25	14.63±0.84*	0.58±0.04	0.56±0.01	0.56±0.09	0.51±0.04
	Y124	21.98±0.21*	17.01±0.89	19.43±0.23**	13.61±0.13**	0.66±0.02*	0.61±0.02*	0.66±0.00**	0.62±0.02*
	Y125	17.91±0.29	16.94±0.77	17.52±0.39*	12.13±0.87	0.54±0.05	0.53±0.02	0.59±0.01*	0.53±0.01

WT2, Yangmai12. The numbers (Y121-Y125) represented different transgenic lines. K0, K1, K2, and K3 showed different levels of potassium application. Data represent means ± SE of three replicates. Data represent means ± SE of three replicates. * and ** indicate that the differences between the means of the transgenic lines and wild type were significant at the P < 0.05 and 0.01 levels, respectively.

Table S5 Transgenic tobacco root morphological parameters under treatment with 0.05 mmol L^{-1} K

Lines	The average height of main stems (cm)	The average number of roots	Total root length (cm)	Total root surface area (cm^2)	Total root volume (cm^3)
WT	10.7±0.6	1572.1±257.3	1188.2±132.5	35.5±2.5	2.5±0.1
#32	17.3±0.6*	2355.1±384.5*	1627.7±91.5**	52.2±4.1*	4.1±0.3*

Data represent means ± SE of three replicates.* and ** indicate that the differences between the means of the transgenic lines and wild type were significant at the P < 0.05 and 0.01 levels, respectively.

Table S6 Soil environment at different field trial locations in 2012 in Zhengzhou

Location	Soil environment before planting		
	Available nitrogen (mg/L)	Available phosphorus (mg/L)	Available potassium (mg/L)
Normal K	15.0	35.8	85.9
Low K	20.6	32.6	53.7

Table S7 Soil environment at different field trial locations in 2013 in Zhengzhou

Location	Soil environment before planting		
	Available nitrogen (mg/kg)	Available phosphorus (mg/kg)	Available potassium (mg/kg)
Normal K	73.87	40.18	189.46
Low K	61.99	9.50	76.53

Table S8 Soil environment at different pot trial locations

Location	Soil environment			
	Available ammonium nitrogen (mg/Kg)	Available nitrate nitrogen (mg/Kg)	Available phosphorus (mg/Kg)	Available potassium (mg/Kg)
Red soil	4.1	0.3	0.9	55.9
Black soil	0.5	10.6	63.3	92.2

Fig. S1

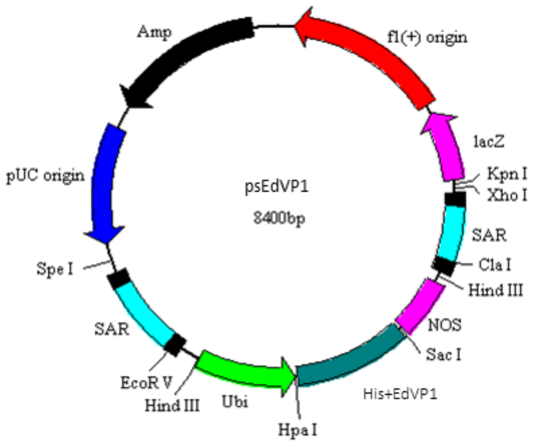

Fig. S1. The map of expression vector of *EdVP1* in transgenic wheat

Fig. S2

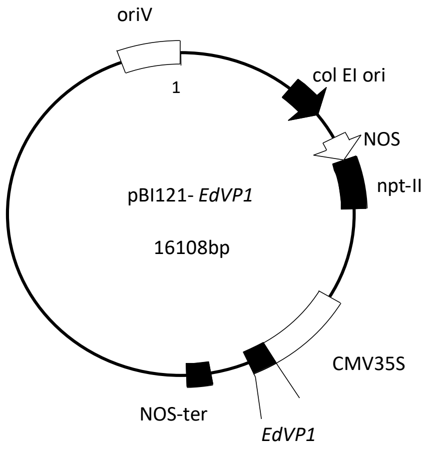

Fig. S2. The map of expression vector of *EdVP1* in transgenic tobacco

Fig. S3

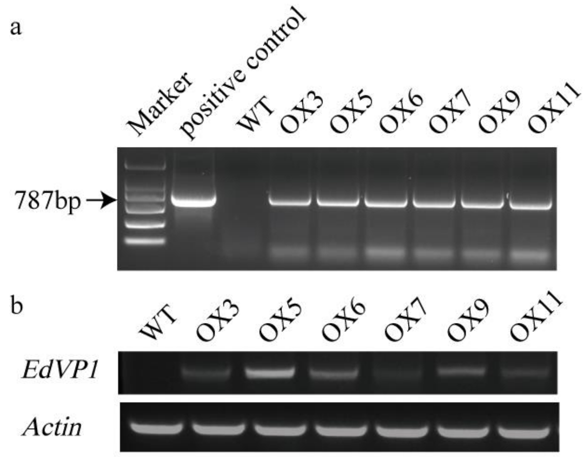

Fig. S3. PCR detection (a) and semi-quantitative RT-PCR (b) results of expressing *EdVP1* in transgenic wheats. Marker, D2000; Positive control, plasmid.

Fig. S4

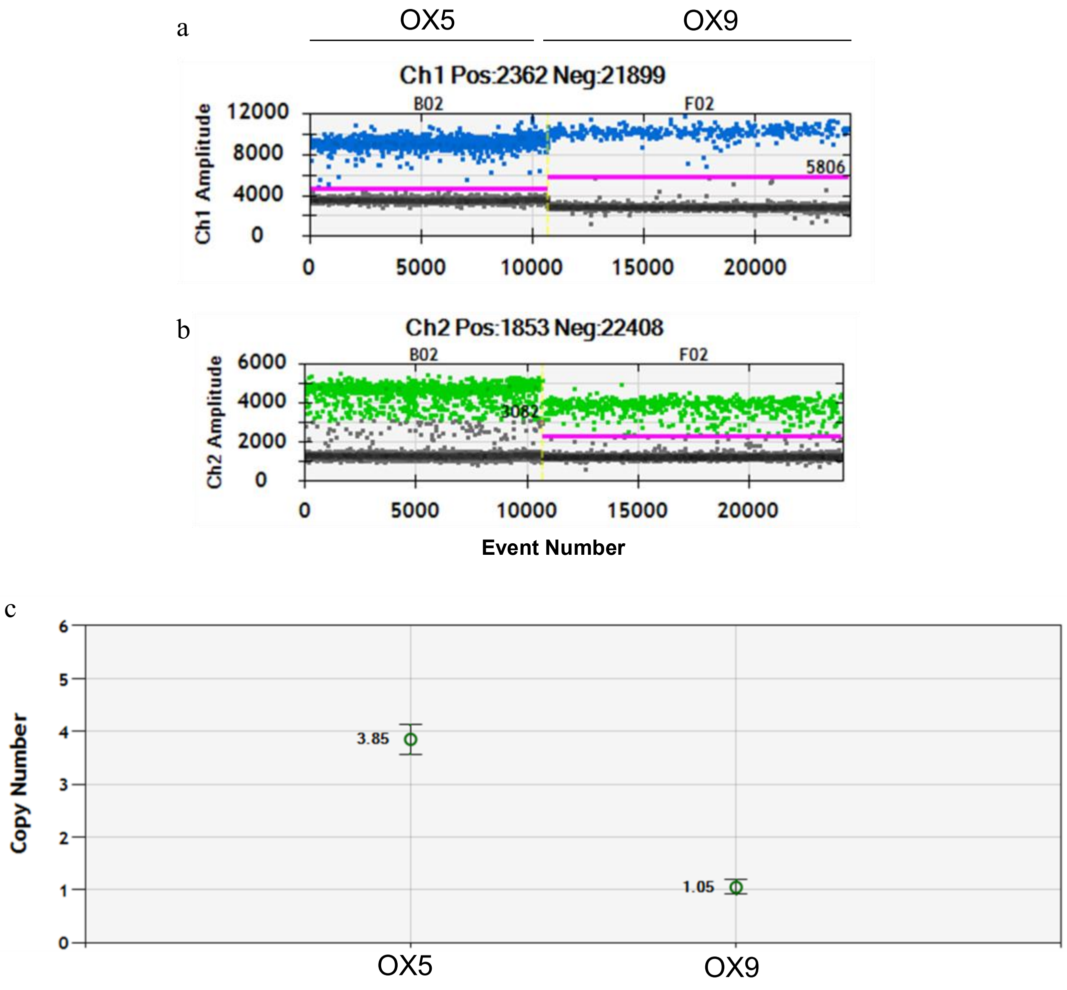

Fig. S4. Droplet digital PCR analysis for determining copy number. (a)-(b) One-dimensional plot of droplets measured for fluorescence signal (amplitude indicated on y-axis) emitted from the endogenous reference gene *PINb-D1b* (positive droplets are blue, a) or the transgene *EdVP1* (positive droplets are green, b). Negative droplets are shown in black. (c) Display of the calculated *EdVP1* copy number in transgenic wheat lines (OX5 and OX9) after copy number variation processing in QuantaSoft™ where the reference gene copy number was set to one, as it is a homozygous gene in two copies per hexaploid genome. The error bars represent the maximum and minimum Poisson distribution for the 95% confidence interval generated by the QuantaSoft™ software.

Fig. S5

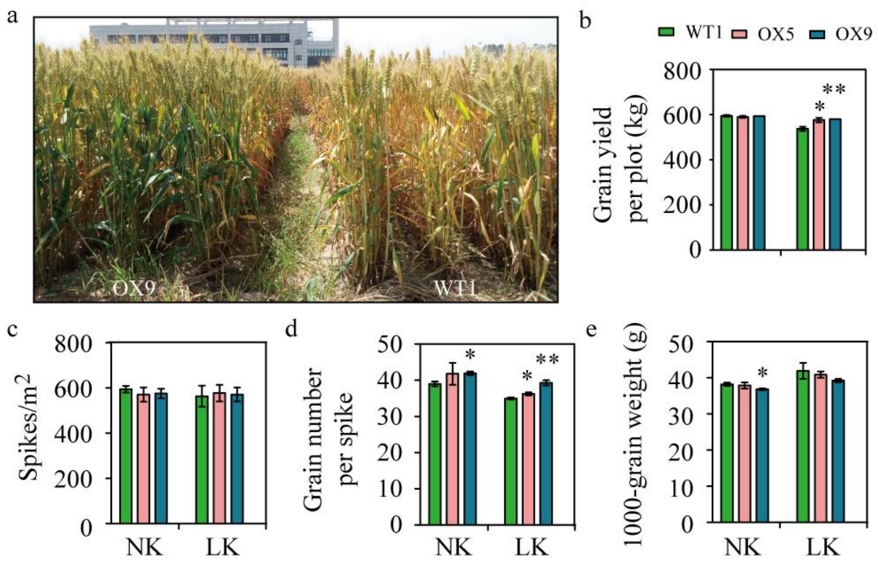

Fig. S5. *EdVP1* transgenic wheat exhibited improvements in a variety of yield performance traits in the 2013-2014 growing season. (a) Field performance of transgenic lines OX9 and the wild type Zheng147 under LK condition. (b) Grain yield per plot (kg). (c) Spikes/m^2. (d) Grain number per spike. (e) 1000-grain weight (g). Data represent means ± SE of three replicates. * and ** indicate that the differences between the means of the transgenic lines and wild type were significant at the $P < 0.05$ and 0.01 levels, respectively.

Fig. S6

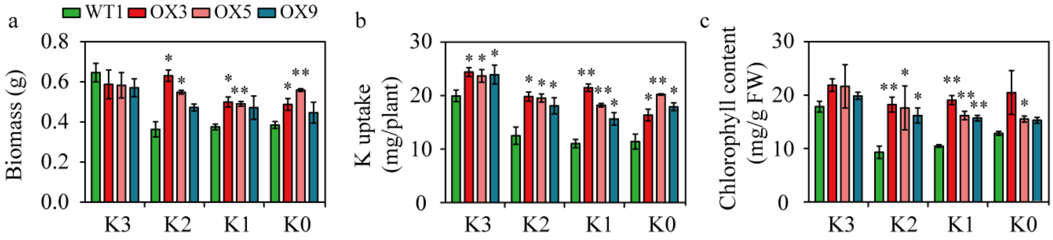

Fig. S6. *EdVP1* overexpression in wheat increased biomass and K uptake in wheat seedlings. (a) Performance of transgenic lines and the wild type Zhengzhou147 under different K treatments in black soil (K0, K1, K2, and K3). K0, no increased potassium level, K1, low potassium level; K2, medium potassium level; K3, high potassium level. (b) Biomass per plant (g). (c) K uptake per plant. (d) Chlorophyll content. Data represent means ± SE of three replicates. * and ** indicate that differences between the means of the transgenic lines and wild type were significant at the P < 0.05 and 0.01 levels, respectively.

Fig. S7

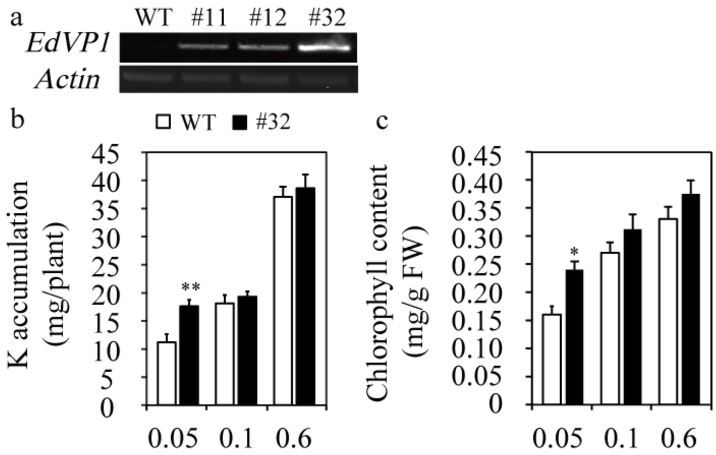

Fig. S7 *EdVP1* overexpression in tobacco plants showed different K accumulation and chlorophyll content in MS medium (containing 0.05 mM, 0.1 mM, and 0.6 mM K). (a) Results of expressing *EdVP1* in transgenic tobacco lines. (b) K accumulation per plant. (c) Chlorophyll content. Data represent means ± SE of three replicates. * and ** indicate that differences between the means of the transgenic lines and wild type were significant at the P < 0.05 and 0.01 levels, respectively.

Fig. S8

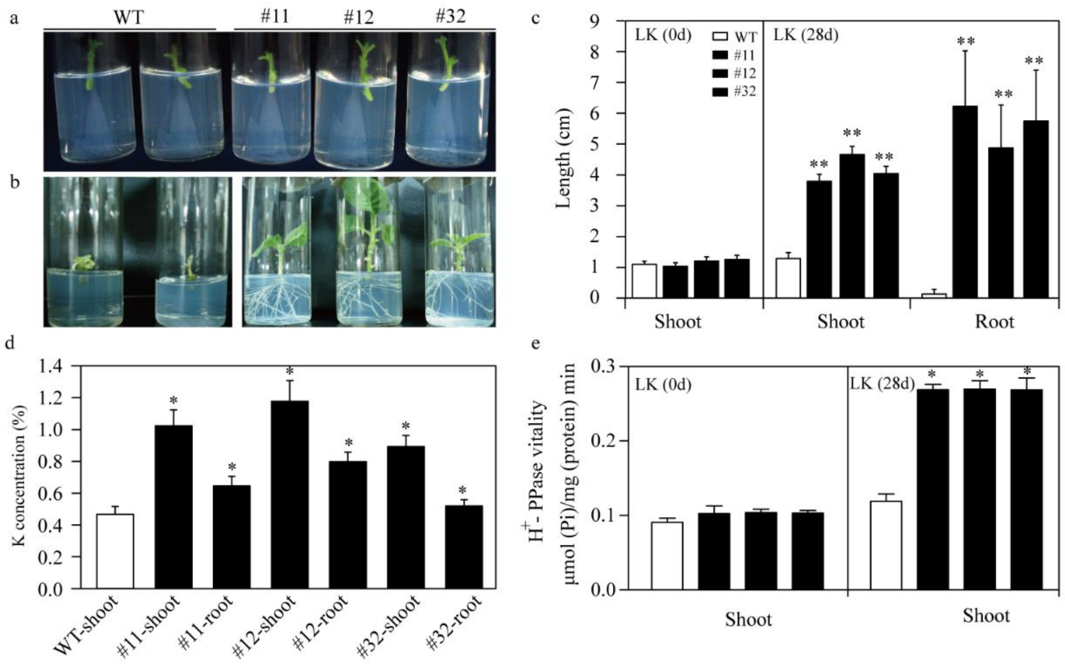

Fig. S8. Overexpression of *EdVP1* improved adaptation to potassium (K) starvation in transgenic tobacco plants. (a) Growth of stem segments before low K treatment. (b) The growth of stem segments was observed after four weeks of LK treatment. (c) The length of shoot and root before and after LK treatment. (d) K concentration in shoot and root under LK treatment. (e) H^+-PPase vitality before and after LK treatment. Data represent means ± SE of three replicates. * and ** indicate that differences between the means of the transgenic lines and wild type were significant at the $P < 0.05$ and 0.01 levels, respectively.

Fig. S9

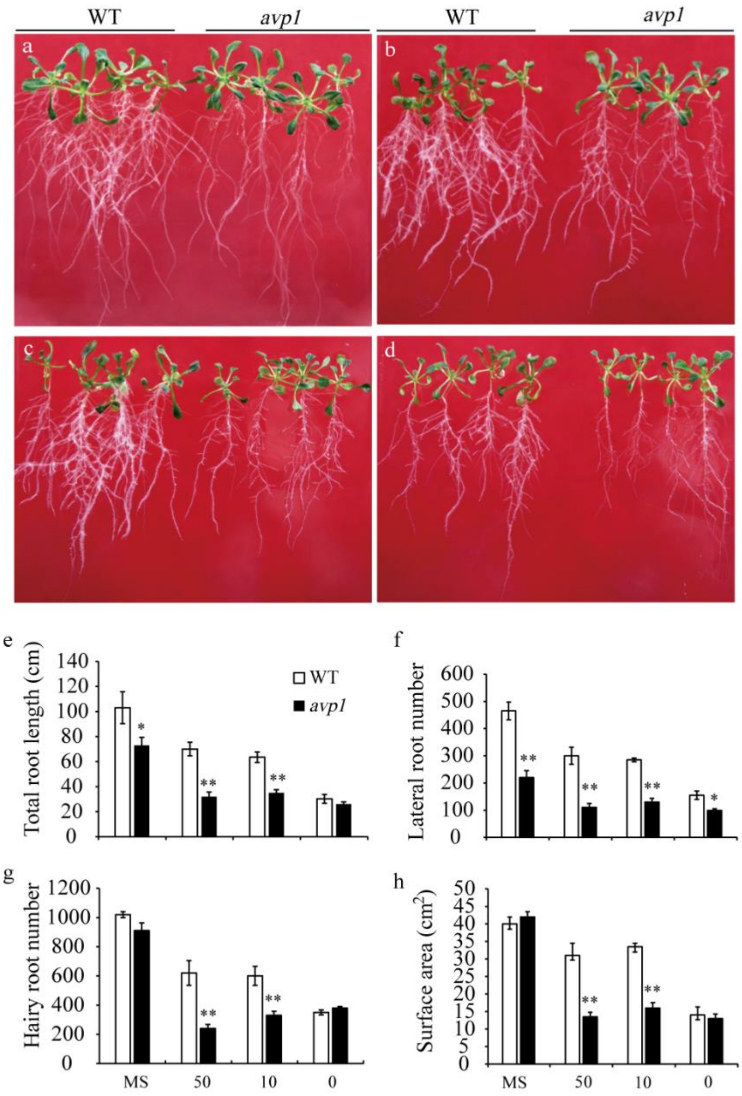

Fig. S9 Phenotypes of the wild type and the *VP1* mutant after different K treatments. (a-d) Performance of shoot and root under different MS medium treatments (a, normal MS medium 1 mM K; b, 50 µM K; c, 10 µM K; and d, 0 µM K). (e) Total root length (cm). (f) Lateral root number. (g) Hairy root number. (h) Root surface area (cm^2). Data represent means ± SE of three replicates. * and ** indicate that differences between the means of the transgenic lines and wild type were significant at the $P < 0.05$ and 0.01 levels, respectively.

Fig. S10

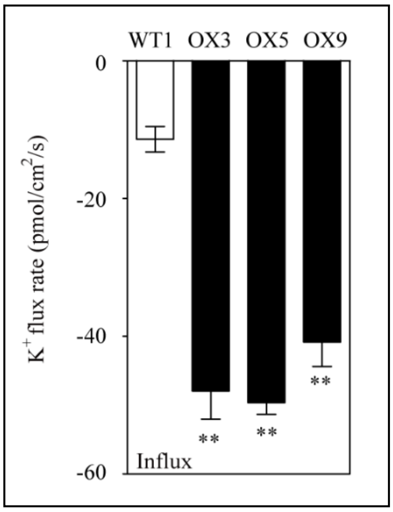

Fig. S10. K^+ flux rate at the root surface of *EdVP1* transgenic wheat.

Fig. S11

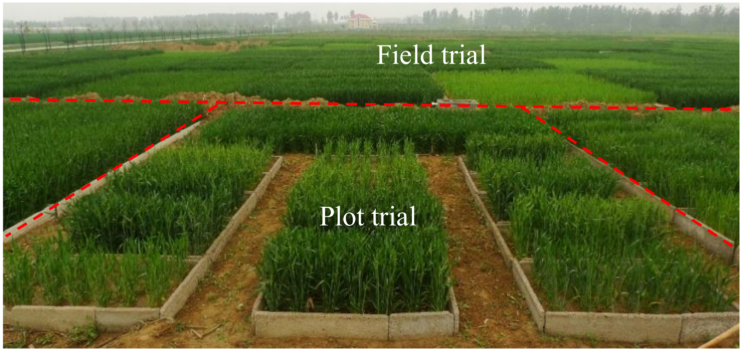

Fig. S11 The plot and field trials were conducted from 2011 to 2014 at the Zhengzhou Fluvo-aquic Soil Fertility and Fertilizer Efficiency Long-Term Monitoring Base.
